# Evaluation of Functional and Clinical Outcomes Following Surgical Repair of Complete Thumb Ulnar Collateral Ligament Injuries in Adults: A Systematic Review Across Diverse Populations

**DOI:** 10.7759/cureus.87053

**Published:** 2025-06-30

**Authors:** Amanuel Kefyalew Assefa, Maysaa N Amin, Rahma Hashish, Khaled Agha Tabari, Shivling S Swami, Alousious Kasagga, Lubna Mohammed, Malik Y Husami, Bantayehu Getachew

**Affiliations:** 1 Orthopaedics and Trauma, University Hospitals of Leicester NHS Trust, Leicester, GBR; 2 Microbiology/Immunology, California Institute of Behavioral Neurosciences and Psychology, Fairfield, USA; 3 Internal Medicine, Sherwood Forest Hospitals NHS Foundation Trust, Nottingham, GBR; 4 Radiology, Queen Elizabeth University Hospital, Glasgow, GBR; 5 Internal Medicine, California Institute of Behavioral Neurosciences and Psychology, Fairfield, IND; 6 Pathology, Peking University, Beijing, CHN; 7 Internal Medicine, Dr. Vizarath Rasool Khan (V.R.K) Women’s Medical College, Hyderabad, IND; 8 Orthopaedics, University Hospitals of Leicester NHS Trust, Leicester, GBR; 9 Public Health, Hosanna College of Health Sciences, Hossana, ETH

**Keywords:** clinical outcome, complete ulnar collateral ligament tear, functional outcome, gamekeeper’s thumb, skier’s thumb, thumb injury, thumb ligament injury, ulnar collateral ligament, ulnar collateral ligament reconstruction

## Abstract

The ulnar collateral ligament (UCL) of the thumb is situated on the inner side of the thumb, near the ulnar aspect of the metacarpophalangeal joint. It plays a crucial role in stabilizing the base of the thumb and contributes to grip strength and overall hand function. UCL injuries account for approximately 86% of all injuries affecting the base of the thumb. Treatment for UCL injuries varies depending on severity. When the UCL of the thumb is completely torn, surgical intervention becomes necessary. Significant gaps in the literature still exist despite the growing number of publications on the surgical treatment of complete thumb UCL tears. Thus, this systematic review synthesized existing research to evaluate the functional and clinical outcomes of surgical treatments for complete UCL ruptures of the thumb across athletes and the general population.

This systematic review was conducted according to the Preferred Reporting Items for Systematic Reviews and Meta-Analyses (PRISMA 2020) guidelines. However, the review protocol was not prospectively registered in a systematic review database such as PROSPERO. Articles published between January 1, 2015 and January 30, 2025 were identified through reputable databases including PubMed, PubMed Central, Europe PubMed Central, ScienceDirect, Embase, and Google Scholar. This review included English-language studies with accessible full texts, conducted on human subjects; 12 studies were reviewed for analysis. The Assessment of Multiple Systematic Reviews 2 (AMSTAR2) critical appraisal tool was used to evaluate all selected systematic reviews, and the Newcastle-Ottawa Scale (NOS) was used to assess the cohort and longitudinal studies.

A total of 335 patients from 12 studies were analyzed, with follow-up ranging from 6 months to 15 years. The studies included retrospective and prospective cohorts, as well as systematic reviews. Surgical techniques varied and included suture anchors, tendon grafts, suture tape augmentation, and novel methods such as ultrasound-welded anchors and U-shaped Kirschner wires. Most studies reported favorable outcomes in joint stability, range of motion, strength, pain relief, and patient satisfaction. Among athletes, return-to-sport rates were high (up to 98.1%), often within 5-8 weeks postoperatively.

Conclusions indicate that surgical management remains the gold standard for complete UCL tears of the thumb, particularly in cases involving Stener lesions, joint instability, or failed conservative treatment. Techniques such as suture anchor repair, tendon graft reconstruction, and internal brace augmentation consistently yield favorable functional and clinical outcomes, including restored strength, reduced pain, and high patient satisfaction. Although earlier intervention generally leads to better results, positive outcomes are observed across diverse populations. This review highlights the need for high-quality randomized controlled trials to standardize surgical indications, techniques, and postoperative care.

## Introduction and background

The ulnar collateral ligament (UCL) of the thumb is situated on the inner side of the thumb, near the ulnar aspect of the metacarpophalangeal (MCP) joint. It plays a crucial role in stabilizing the base of the thumb and contributes to grip strength and overall hand function [1,2].

UCL injuries are responsible for approximately 86% of all injuries affecting the base of the thumb [3]. This common injury is estimated at around 50 cases per 100,000 emergency department visits [4]. Injury to the UCL of the thumb's MCP joint can occur as a result of excessive abduction and extension forces applied to the joint [5]. Thumb UCL injuries are classified into three grades of increasing severity: Grade 1 represents a mild sprain with no ligament tearing, Grade 2 involves a partial ligament tear, and Grade 3 signifies a complete ligament rupture [6].

A complete rupture of the UCL, frequently caused by sudden injury or overuse, is known as either "gamekeeper's thumb" or "skier's thumb," depending on the mechanism of injury. If left untreated, these tears can severely limit thumb function. While partial tears might heal without surgery, complete tears usually require surgical repair to re-establish stability and restore proper thumb function [1,7]. Non-surgical approaches often fail to restore joint stability, resulting in persistent instability and reduced thumb functionality. Studies have shown that non-surgical treatment frequently proves unsuccessful and often necessitates delayed surgical intervention, whereas initial surgical repair is associated with better clinical outcomes [8].

Treatment for UCL injuries of the thumb varies with severity. Grade 1 and 2 injuries, involving sprains and partial tears, usually require four weeks of immobilization followed by rehabilitation to regain motion and strength [5]. When the UCL is completely torn, surgical intervention becomes necessary. Surgical options include primary repair of the existing ligament, reconstruction using autografts (such as tendon grafts), and newer techniques like suture tape augmentation. These approaches aim to restore joint stability and prevent long-term complications such as chronic instability or osteoarthritis. Despite advancements in surgical techniques, there is still debate about the optimal surgical approach for different patient populations, including athletes, pediatric patients, and individuals with chronic injuries. Research has demonstrated that both immediate repair and delayed reconstruction can yield effective outcomes for chronic injuries, with comparable long-term results in terms of functional recovery and complication rates [9-11].

According to the majority of published research, patients who undergo surgery after a complete UCL tear generally recover well and experience few serious aftereffects. A study on sports-related injuries reported a 10.3% postoperative complication rate and a 98.1% overall successful return to play following surgery, without a discernible decline in performance [12]. While splint therapy was found to be effective in one trial, 15% of patients from a general population with a complete UCL rupture experienced ongoing pain and instability, requiring surgical reconstruction after 12 weeks [13].

Significant gaps in the literature still exist despite the growing number of publications on the surgical treatment of complete thumb UCL tears. Many current studies have limited sample sizes, lack standardized outcome measures, or focus on specific demographics, such as professional athletes. These limitations, such as small sample sizes, short follow-up periods, and a predominant focus on athletic populations, restrict the applicability of current findings to the broader patient population. This limits clinicians’ ability to confidently generalize treatment outcomes to non-athlete individuals, thereby underscoring the need for a comprehensive review of evidence to support clinical decision-making and improve patient care.

This systematic review synthesizes existing research to evaluate the functional and clinical outcomes of surgical treatments for complete UCL ruptures of the thumb across various patient groups. Specifically, the review assesses outcomes such as grip strength, range of motion at the MCP joint, pain levels, time to return to daily activities or sport, patient-reported satisfaction, and postoperative complications. The data analysis aims to provide insight into the practical effectiveness of different surgical techniques and rehabilitation approaches, with a particular emphasis on indicators related to returning to normal function. By synthesizing evidence on key clinical and functional outcomes, including grip strength, range of motion, time to return to activity, pain levels, and joint stability, this review supports healthcare professionals in designing individualized treatment plans and enhancing recovery outcomes for their patients.

## Review

Methods

This systematic review was organized and reported according to the PRISMA 2020 guidelines, ensuring clarity and completeness [14]. However, the review protocol was not prospectively registered in a systematic review database such as PROSPERO.

Database and Search Strategy

We conducted a thorough literature search to identify relevant studies and ensure a comprehensive review. Our search focused on publications from January 1, 2015, to January 30, 2025, using several key databases, including PubMed (with Medical Subject Headings [MeSH]), PubMed Central, Europe PubMed Central, ScienceDirect, Embase, and Google Scholar. We used a combination of targeted keywords such as: "Ulnar collateral ligament, collateral ligament injuries, Metacarpophalangeal Joint, Injuries, surgery, Thumb/injuries, Collateral ligament", ("ulnar collateral ligament tear" OR "UCL tear" OR "gamekeeper's thumb" OR "skier's thumb" OR "chronic UCL tears" OR "acute vs. delayed repair") AND ("thumb MCP joint" OR "metacarpophalangeal joint") AND ("functional outcomes" OR "clinical outcomes"). To ensure accuracy and relevance, we adapted these search terms based on the specific indexing systems of each database.

In addition to database searches, we manually examined citations to ensure thoroughness. Data extraction was performed independently by two reviewers. Study quality was independently assessed using the Newcastle-Ottawa Scale (NOS) by two authors (Assefa A and Amin M). All co-authors contributed to the review process through collaboration in study selection, manuscript review, and refinement of content and structure. Regular weekly Zoom meetings enabled collaborative discussions on progress, results, and strategic changes. The detailed search strategies and identified articles are recorded in Table 1.

**Table 1:** Articles identified from each database. UCL: Ulnar Collateral Ligament; MCP: Metacarpophalangeal; MeSH: Medical Subject Headings; PMC: PubMed Central; PRISMA: Preferred Reporting Items for Systematic Reviews and Meta-Analyses.

Database	Keywords	Search Strategy	Filters	Search Result
PubMed (MeSH/Medline/PMC)	Ulnar collateral ligament, collateral ligament injuries, Metacarpophalangeal Joint, injuries, surgery, thumb injuries, collateral ligament	(("Ulnar Collateral Ligament" OR "Ulnar Collateral Ligament Injury" OR "Chronic UCL Tears" OR "Acute versus Delayed Repair" OR "Thumb UCL Tear" OR "Gamekeeper’s Thumb" OR "Skier’s Thumb" OR "Thumb Ligament Injury" OR "Metacarpophalangeal Joint Injury" OR "Complete Ulnar Collateral Ligament Tear") AND ("UCL Repair" OR "UCL Reconstruction" OR "Surgical Treatment" OR "Suture Anchor Repair" OR "Tendon Graft Reconstruction" OR "Ligament Repair" OR "Primary Repair" OR "Reconstruction Surgery" OR "Augmentation") AND ("Clinical Outcomes" OR "Functional Outcomes" OR "Joint Stability" OR "Healing Rate" OR "Complication Rate" OR "Re-rupture" OR "Infection Rate" OR "Radiographic Outcomes" OR "Range of Motion" OR "Grip Strength" OR "Pinch Strength" OR "Patient-Reported Outcomes" OR "Return to Sport" OR "Return to Work" OR "Hand Function" OR "Postoperative Results" OR "Surgical Success" OR "Pain Scores")) NOT ("Elbow UCL Reconstruction" OR "Tommy John Surgery" OR "Ulnar Collateral Ligament of the Elbow" OR "Elbow Surgery")	Last 10 years, full text, English language, human	43
Europe PMC	Complete ulnar collateral ligament tear of thumb	("Ulnar Collateral Ligament Tear" OR "UCL Tear" OR "Gamekeeper's Thumb" OR "Skier's Thumb") AND ("Thumb MCP Joint" OR "Metacarpophalangeal Joint" OR "Thumb Joint") AND ("Functional Outcome*" OR "Clinical Outcome*" OR "Treatment Outcome*" OR "Range of Motion" OR "Grip Strength" OR "Pain Score" OR "Disability" OR "Return to Function") AND (FIRST_PDATE:[2015 TO 2025])	Last 10 years, full text	26
Embase	Ulnar collateral ligament tear, thumb injury	(Complete ulnar collateral tear OR ulnar collateral ligament injury) AND ("Clinical Outcomes" OR "Functional Outcomes")	Full text online, open access, scholarly and peer-reviewed, last 5 years, English	131
Google Scholar	Complete UCL tear, thumb, metacarpophalangeal, chronic UCL tears, acute vs. delayed repair	("Ulnar Collateral Ligament Tear" OR "UCL Tear" OR "Gamekeeper's Thumb" OR "Skier's Thumb" OR "Chronic UCL Tears" OR "Acute vs. Delayed Repair") AND ("Thumb MCP Joint" OR "Metacarpophalangeal Joint") AND ("Functional Outcomes" OR "Clinical Outcomes")	Last 10 years	96
ScienceDirect	Complete ulnar collateral ligament tear, thumb, chronic UCL tears, acute vs. delayed repair	Complete ulnar collateral ligament tear, thumb OR chronic UCL tears, acute vs. delayed repair	Last 10 years	211

The risk of bias for non-randomized studies was assessed using the NOS. Two independent reviewers assigned scores based on predefined NOS criteria. Any discrepancies were resolved through discussion or consultation with a third reviewer. The bias assessment results were presented in a structured format, ensuring transparency in evaluating the reliability of the included studies.

Inclusion and Exclusion Criteria

This systematic review included peer-reviewed articles published in English between 2015 and 2025, comprising cohort studies and systematic reviews. Inclusion criteria required that all articles provide unrestricted access to their full text. Furthermore, only studies incorporating both male and female participants were considered for analysis.

The review included studies focusing on patients with surgically managed, complete UCL tears of the thumb, diagnosed clinically and through imaging. To be eligible, studies had to report at least one functional outcome measure (e.g., grip strength, range of motion (ROM), patient-reported outcomes) and at least one clinical outcome measure (e.g., pain scores, joint stability), with a minimum postoperative follow-up duration of six months, as this duration provides sufficient time to assess functional recovery. Exclusion criteria included studies published more than ten years prior, those not in English, non-human studies, and articles without available full text. Additionally, studies involving patients with partial UCL tears, other thumb injuries (e.g., fractures), non-operative treatments, or those failing to report both functional and clinical outcomes were excluded.

Selection Process 

All relevant articles were imported into EndNote (Clarivate, Philadelphia, PA), and duplicate entries were removed. Two reviewers independently conducted title and abstract screening, followed by full-text assessment to determine study eligibility. Full-text availability was confirmed for the selected articles. The most relevant papers then underwent further evaluation based on the predefined inclusion and exclusion criteria. Finally, studies that met all eligibility requirements were included in the analysis.

Quality Appraisal and Data Collection Process

The quality appraisal of the selected studies was conducted using tools appropriate to each study design. For systematic reviews, the AMSTAR 2 tool was used to evaluate methodological quality, while the NOS was applied to assess cohort and longitudinal studies. Two co-authors independently performed the quality assessments. After gathering all relevant articles, each co-author contributed their evaluation data for the systematic review. The primary focus of this study was to assess the functional and clinical outcomes following surgical repair of complete thumb UCL injuries in adults across various populations.

Results

Study Selection

Prior to April 30, 2025, a comprehensive search was conducted across PubMed, Europe PubMed Central, Embase, Google Scholar, and ScienceDirect, yielding 507 relevant studies. After removing 67 duplicates using EndNote, 470 unique articles remained for detailed review. Titles and abstracts were screened, and full texts were assessed for availability, resulting in 50 articles shortlisted for retrieval, while 420 were excluded. These shortlisted studies were then further evaluated against the inclusion and exclusion criteria, leading to the removal of 27 articles. Ultimately, 23 studies were assessed for eligibility and quality using appropriate appraisal tools tailored to each study type. Any disagreements between reviewers during the quality assessment process were resolved through discussion and consensus. Quality assessment using specialized instruments identified 12 high-quality studies scoring above 70%, comprising 10 cohort studies and 2 systematic reviews, which were included in the final analysis. A meta-analysis was not performed due to substantial heterogeneity across the included studies.

**Figure 1:**
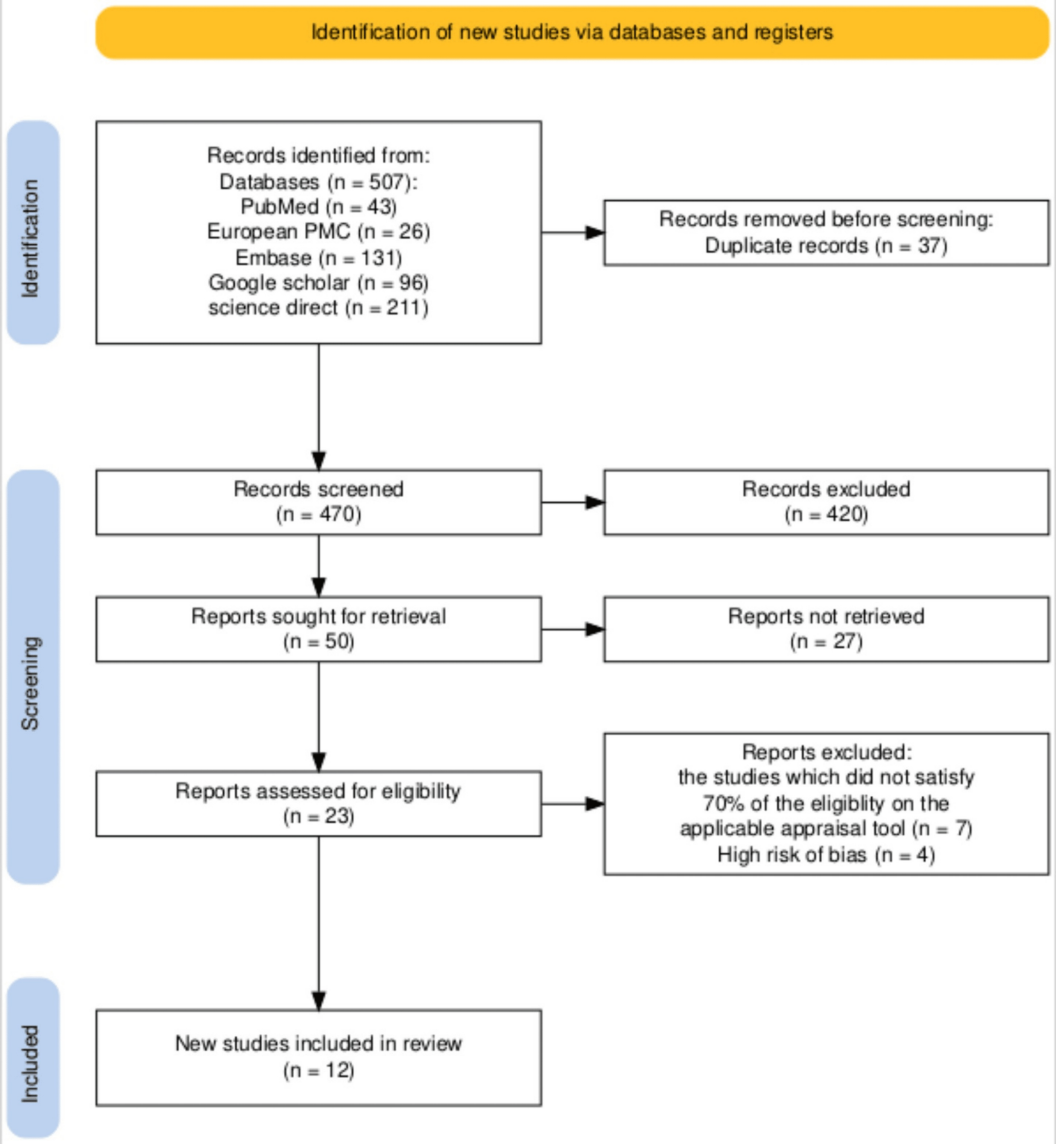
PRISMA diagram illustrating the process of article selection. PRISMA: Preferred Reporting Items for Systematic Reviews and Meta-Analyses; PMC: PubMed Central.

The PRISMA flowchart, which illustrates the selection and screening process, is represented in Figure 1.

Quality Assessment

The quality of the 10 included cohort and longitudinal studies was evaluated using the Newcastle-Ottawa checklist [15]. The assessment criteria are presented in Table 2.

**Table 2:** Newcastle-Ottawa cohort checklist used for quality assessment of the included non-randomized cohort and longitudinal studies. Based on the Newcastle-Ottawa Scale (NOS) for cohort studies, a study earns one star (★) for meeting the criteria in questions 2 and 4 under the Selection domain, and question 7 under the Outcome domain. Two stars (★★) may be awarded for questions 1 and 3 in the Selection category, question 5 under Comparability, and questions 6 and 8 in the Outcome domain if the study fully meets the specified criteria. A study is considered high quality if it scores 3-4 stars in Selection, 1-2 stars in Comparability, and 2-3 stars in Outcome. Fair quality applies to studies with 2-3 stars in Selection, 1-2 in Comparability, and 2-3 in Outcome. Poor quality refers to those scoring 0-1 in Selection, 0 in Comparability, and 0-1 in the Outcome domain.

Authors (Year)	Study Type	Selection Q1	Q2	Q3	Q4	Comparability	Outcome	Total Score (9)	Quality
Kastenberger T et al. (2021) [16]	Prospective Cohort	★		★	★	★	★★★	8	High
Ayık Ö and Demirel M (2022) [17]	Retrospective Case Series	★★		★	★	★	★★★	8	High
Delma S et al. (2022) [18]	Retrospective Cohort	★★	★	★	★	★	★★★	8	High
Barrera-Ochoa S et al. (2024) [19]	Retrospective Cohort	★★	★	★	★	★	★★★	8	High
Gibbs DB and Shin SS (2020) [20]	Retrospective Case Series	★★		★	★	★	★★★	8	High
Christensen T et al. (2016) [21]	Retrospective Cohort	★★		★	★	★	★★★	8	High
Legerstee IW et al. (2023) [6]	Prospective Cohort	★★		★	★	★	★★★	8	High
Jack RA 2nd et al. (2018) [22]	Retrospective Cohort	★	★	★	★	★★	★★★	8.5	High
Legerstee IW et al. (2024) [23]	Prospective Cohort	★★		★	★	★	★★★	8	High
Ma S et al. (2024) [24]	Retrospective Cohort	★	★	★	★	★	★★★	8	High

We used the AMSTAR 2 to assess the quality of the two included systematic reviews (Table 3) [25].

**Table 3:** AMSTAR 2 score used for quality assessment of the included systematic reviews. AMSTAR 2: Assessment of Multiple Systematic Reviews 2.

Author(s)	Study Design	AMSTAR 2 Score (out of 16)
Mikhail M et al. (2018) [26]	Systematic review (3 RCTs and 3 retrospective comparative studies)	12/16
Allahabadi S et al. (2023) [12]	Systematic review (narrative synthesis)	12/16

Study Characteristics 

A summary of the main characteristics of the observational studies included in the analysis is presented in Table 4.

**Table 4:** Summary of the main characteristics of the observational studies included in the analysis. UCL: Ulnar Collateral Ligament; MCP/J: Metacarpophalangeal (Joint); BLR: Biological Ligament Reconstruction; NBLR: Non-Biological Ligament Reconstruction; MLB: Major League Baseball.

Author and Year	Study Design	Inclusion Criteria	Surgical Technique Used	Sample Size	Outcome
Ayık Ö and Demirel M (2022) [17]	Retrospective study	Studies in which patients underwent surgical reconstruction of both the ulnar collateral ligament and dorsal joint capsule for chronic UCL injuries with volar subluxation, with a minimum follow-up of 12 months, complete medical and radiological data, and patients with mental status compatible with treatment.	Novel suture reconstruction	7	The mean preoperative flexion deficit increased from 10.7° to 31.4° (P = 0.034). Key-pinch strength improved from 33.2% to 10.2% (P < .001), and hand grip strength improved from 18.8% to 6.4% (P < .001) at final follow-up.
Delma S et al. (2022) [18]	Retrospective study	Patient charts of those who underwent operative UCL repair between 2006 and 2013. Patients with more than 8 weeks between injury and surgery were classified as chronic cases.	Primary repair with suture anchors and local soft tissue advancement	36	Mean postoperative QuickDASH scores were similar for both acute and chronic groups: 3.2 and 3.6, respectively (P = 0.42). Both groups showed comparable pain levels, satisfaction, and return-to-activity rates.
Barrera-Ochoa S et al. (2024) [19]	Retrospective study	Patients with isolated, complete, symptomatic chronic thumb MCP joint UCL injuries who received either static BLR with tendon graft or NBLR using FiberTape, with ≥3 years of follow-up.	Comparison of biological tendon grafts vs non-biological implants (e.g., anchors, screws)	42	Non-biological UCL reconstruction showed similar short-term outcomes to biological methods. ROM and strength were comparable. Group outcomes: 102% vs 103% grip strength, 84% vs 89% pinch strength.
Kastenberger T et al. (2021) [16]	Prospective cohort study	Patients ≥18 years scheduled for surgery for gross ulnar-sided MCP instability of the thumb using a 2.3 mm Fiji Anchor® (SportWelding® GmbH, Schlieren, Switzerland).	BoneWelding® Fiji® Anchor with ultrasound-welded polymer anchors	24	Final follow-up showed MCP motion near normal (49.3° ±11.7°), Kapandji score 9.7. Grip/pinch strength was 83-101% of the unaffected hand. Low pain scores; DASH = 5.0; PRWE = 4.1. 81% were very satisfied.
Christensen T et al. (2016) [21]	Retrospective study	Patients with chronic, complete thumb UCL injuries treated before Jan 1998, ≥15 years of follow-up, and no preoperative MCP osteoarthritis.	Suture repair (no grafts)	21	Local tissue repair may be a viable long-term alternative to reconstruction. Most patients eventually developed osteoarthritis.
Legerstee IW et al. (2023) [6]	Prospective cohort study	Patients undergoing open surgical repair for complete thumb UCL rupture (Dec 2011 - Feb 2021).	Open surgical repair with suture anchors; some with Kirschner wires or bone tunnels	76	Patient-reported outcomes improved at 3 and 12 months. No correlation between injury-to-surgery interval and MHQ scores.
Jack RA 2nd et al. (2018) [22]	Cohort study	Athletes on MLB rosters with confirmed thumb UCL repair (via ≥2 sources).	Suture anchors and/or tape	21	All MLB players returned to sport. Mean in-season return: 8 weeks. Career length and games played were comparable to controls. Infielders had lower post-op WAR than pre-injury, but not vs controls.
Allahabadi S et al. (2023) [12]	Systematic review	English-language studies on surgical treatment of thumb UCL injuries in athletes (all levels).	Anchors, suture tape, tendon grafts	23	High return-to-play rate (98.1%) with low complications (10.3%) and no decline in performance. Mean MINORS score = 9.4.
Legerstee IW et al. (2024) [23]	Retrospective cohort study	Patients undergoing tendon autograft reconstruction (Dec 2011 - Feb 2021).	Tendon autograft reconstruction	31	84% regained MCP stability. Pinch strength improved from 5.2 kg to 6.4 kg. MHQ scores remained unchanged in patients with persistent instability.
Mikhail M et al. (2024) [26]	Systematic review	Studies on acute complete ruptures of the thumb UCL at MCPJ in adults.	Biological reconstruction (tendon grafts) vs non-biological (anchors, tape)	6	No prospective studies compared surgical vs non-surgical management for acute complete UCL rupture.
Gibbs DB and Shin SS (2020) [20]	Retrospective study	Competitive athletes (high school to professional) with acute complete UCL tears managed by suture tape augmentation.	UCL repair with suture tape augmentation	18	All athletes returned to sport, with in-season return <5 weeks. Augmentation reduced immobilization, enhanced thumb mobility.
Ma S et al. (2024) [24]	Retrospective study	Criteria: (1) ulnar avulsion fractures of thumb P1 base; (2) positive ulnar deviation stress test; (3) ≤1-week-old fresh fracture; (4) fragment ≤30% of joint surface; (5) >1 mm displacement or rotational deformity; (6) closed fractures.	U-shaped Kirschner wire fixation	30	Both techniques reduced pain and improved ROM. Group B had better outcomes in tip-pinch and grip strength; fewer complications were noted.

Discussion

This systematic review investigated the surgical treatment of complete UCL tears in the thumb, with a focus on functional and clinical outcomes across various populations. The results indicate that surgical intervention generally leads to positive outcomes in terms of stability and grip strength. However, the most effective surgical technique and rehabilitation protocol continue to be debated, as factors such as injury duration, patient age, and physical activity level influence outcomes.

Surgical Management

Surgical intervention remains the standard approach for managing complete UCL tears of the thumb, particularly in cases complicated by Stener lesions, chronic instability, or failure of conservative management. Various techniques have been employed, including suture anchor fixation, transosseous suturing, tendon graft reconstruction, and, more recently, internal brace augmentation. Among these, suture anchor repair is one of the most frequently utilized methods. Multiple studies suggest that this technique provides reliable restoration of joint stability, grip strength, and ROM, particularly when surgery is performed early after injury [17,21]. Complication rates were generally low, and patients often reported high postoperative satisfaction. Early surgical intervention using suture anchors may offer the best functional and recovery outcomes. Importantly, the choice of surgical technique was primarily guided by the surgeon’s expertise, the duration of the injury, and patient-specific considerations such as activity level, rather than by definitive evidence favoring one approach over another.

Reconstruction, rather than primary repair, is often necessary in chronic UCL injuries where the ligament is of poor quality or significantly retracted. One study highlighted the effectiveness of autologous tendon grafts, particularly in younger and more physically active patients, demonstrating favorable outcomes in restoring thumb stability and function [16]. Another investigation noted that delayed intervention in chronic UCL cases frequently necessitated more complex reconstructive procedures, which were associated with prolonged rehabilitation and slightly inferior functional outcomes compared to acute repairs [22]. Additionally, a retrospective study evaluating tendon autograft reconstruction reported significant improvements in both objective clinical metrics and subjective patient-reported outcomes [23]. These findings suggest that although reconstructive methods are effective, they are most appropriate when early intervention is not feasible or prior surgical procedures have failed. Therefore, clinicians should prioritize early diagnosis and timely surgical management to avoid the need for more complex reconstructions, particularly in high-demand patients. Moreover, the development of standardized criteria to guide the decision between primary repair and reconstruction could help optimize functional outcomes and resource utilization.

A retrospective chart review compared biological ligament reconstruction (BLR) and non-biological ligament reconstruction (NBLR) techniques for treating complete UCL injuries. Biological reconstruction involves using autologous tendon grafts, typically harvested from the palmaris longus or flexor carpi radialis, to biologically replace the damaged ligament, allowing for natural tissue integration and healing. In contrast, non-biological reconstruction employs synthetic materials, such as 2-mm suture tape and knotless anchors, to mechanically stabilize the joint without using donor tissue. The NBLR technique provides immediate structural support, facilitating earlier postoperative mobilization, often as soon as one week after surgery [19]. The study reported comparable outcomes between the two methods regarding ROM, grip strength, and patient satisfaction. Notably, NBLR was associated with significantly shorter operative times and suggested a quicker return to daily activities. These findings highlight internal bracing as a viable alternative to traditional graft-based reconstruction, particularly when early mobilization and reduced surgical time are prioritized. Further prospective studies are warranted to determine whether NBLR offers sustained long-term advantages over BLR in both acute and chronic UCL injury contexts.

Although multiple surgical approaches exist, current evidence underscores the importance of timely surgical intervention customized to the injury’s chronicity and severity. The heterogeneity of surgical methods, such as suture anchor repair, tendon graft reconstruction, suture tape augmentation, and use of Kirschner wires, complicates efforts to establish uniform treatment guidelines. These approaches vary substantially in surgical complexity, postoperative rehabilitation requirements, and the depth of existing evidence, making it difficult to evaluate their relative effectiveness. Such methodological inconsistencies likely contribute to outcome discrepancies between studies, emphasizing the need for rigorous randomized controlled trials (RCTs) that directly compare these techniques under standardized conditions.

*Functional and Clinical Outcomes* 

Functional and clinical outcomes are key indicators of surgical success in treating complete UCL tears of the thumb, as they directly impact a patient's ability to return to daily activities, work, or sports. Most studies report favorable postoperative results, including improved grip strength, pinch strength, and subjective patient satisfaction [16,17,19,21].

A retrospective analysis examining long-term outcomes in chronic cases noted that even with a high prevalence of radiographic osteoarthritis (88%), patients experienced excellent subjective recovery [21]. The study reported a mean DASH score of 5.9 and a mean VAS pain score of 0.6, indicating minimal disability and discomfort. Notably, 92% of patients rated their thumb function as nearly normal, and all participants expressed satisfaction with the surgical outcome. Objective measurements further supported these perceptions, with grip and pinch strength averaging 97% and over 90%, respectively, of the contralateral hand. While clinical assessment revealed some residual laxity in 67% of cases, only 15% of patients reported any sense of instability. This disparity suggests that minor residual laxity may not significantly compromise perceived function or quality of life. These findings underscore the importance of patient-centered outcome measures and suggest that functional recovery and patient satisfaction may be more meaningful indicators of surgical success than isolated clinical metrics of joint stability.

A systematic review identified compelling evidence favoring early mobilization protocols following surgical repair of UCL injuries. Rocchi et al. conducted a RCT involving 40 patients with acute complete UCL ruptures, all of whom underwent surgical repair and were subsequently randomized to receive either traditional thumb spica immobilization (which restricts the metacarpophalangeal (MCP) joint) or a modified orthosis allowing early MCP joint movement [26]. At the 12-month follow-up, patients in the early mobilization group exhibited significantly better outcomes, including greater MCP joint ROM (SMD -3.69), improved functional scores on the Dreiser index (SMD 1.65), and lower pain levels on the visual analog scale (VAS) (SMD 1.53). Interestingly, no significant difference was observed in tip pinch strength between the groups at any time, suggesting that early motion enhances function and comfort without compromising mechanical strength.

Another systematic review across 11 studies involving 311 athletes reported an overall return-to-play (RTP) rate of 98.1%, with nearly all athletes resuming their pre-injury level of performance. Pre-injury performance in this context typically referred to the athlete’s ability to return to the same level of competition, sport-specific function, and playing time or workload as prior to the injury. The average time to RTP across the included studies ranged from 2.5 to 6 months, depending on the sport and surgical technique [12]. Notably, elite athletes, including Major League Baseball (MLB) and National Football League (NFL) players, did not experience any measurable decline in competitive performance following surgery, as reported in studies by Jack et al. and Sochacki et al. These findings reinforce that surgical management of UCL injuries can lead to excellent long-term outcomes when paired with evidence-based rehabilitation protocols. The most commonly employed surgical techniques in these studies included UCL reconstruction using autografts, particularly the modified Jobe technique and docking technique, which are well-established procedures aimed at restoring ligament stability and optimizing athletic performance. Given these results, clinicians may consider early controlled mobilization in appropriate patients to optimize functional recovery and patient comfort without compromising strength or return-to-play timelines, which typically ranged from 2.5 to 6 months. A retrospective study showed impressive short-term results after surgically reconstructing both the UCL and the dorsal joint capsule (DJC) in patients with chronic UCL injuries and volar subluxation, a condition in which the proximal phalanx shifts abnormally toward the palm, indicating not only UCL insufficiency but also dorsal capsular laxity. Because the DJC plays a key role in preventing palmar displacement, its involvement necessitates dual reconstruction to restore MCP joint stability. This innovative approach significantly enhanced clinical and functional outcomes. Notably, pain, as measured by the VAS, dropped from an average of 5.7 to 0.57 post-surgery (P < .001). Additionally, there was a substantial improvement in upper limb function, with Quick-DASH scores improving from 31.8 to 3.2 (P < .001) [17]. These findings suggest that combined ligamentous and capsular reconstruction may provide superior symptom relief and functional recovery in complex, chronic cases involving joint instability.

Similarly, another retrospective study compared traditional Kirschner wire fixation (Group A) with U-shaped Kirschner wire fixation (Group B). Both techniques significantly improved postoperative ROM and reduced pain; however, U-shaped wire fixation demonstrated superior functional recovery in specific parameters. Specifically, patients in Group B achieved higher scores in tip pinch (92.2% vs. 81.86%, P < .001) and grip strength (91.92% vs. 82.39%, P = .008), indicating a more favorable restoration of thumb strength and utility [24]. These findings suggest that subtle technical modifications in fixation strategy can enhance functional restoration, particularly in tasks requiring fine motor control and strength. Such evidence supports the continued refinement of surgical techniques to optimize individualized outcomes, especially in complex or chronic UCL injury cases.

The systematic review findings highlight evidence-based considerations for surgical decision-making in complete thumb UCL tears. Key indications for surgery include persistent valgus laxity >30° or absence of a firm endpoint on stress testing, both of which correlate with functional impairment. Early surgical intervention is often favored for high-demand patients (e.g., athletes, manual laborers) to optimize recovery timelines and prevent chronic instability. For acute tears with good tissue quality, suture anchor fixation is the preferred surgical technique, as it delivers strong outcomes and is associated with fewer complications [16,21,22]. In contrast, chronic or neglected tears typically necessitate ligament reconstruction with tendon grafts or synthetic augmentation due to scarred or retracted tissue [12,24,26]. Postoperative rehabilitation remains debated, but recent studies support early controlled movement over prolonged immobilization to enhance functional recovery while maintaining joint stability [6,19,23]. Collectively, these findings support a personalized surgical and rehabilitation approach that considers injury duration and chronicity. Acute UCL injuries may benefit from prompt surgical repair or bracing with early mobilization, while chronic injuries, often involving ligament degeneration or capsular insufficiency, may require more complex reconstructive procedures. In both cases, tissue quality and patient activity level are critical to achieving optimal long-term outcomes.

Patient-Reported Outcomes

Both the recent prospective study by Legerstee et al. (2023) and the long-term follow-up by Christensen et al. (2016) contribute valuable insights into the sustained effectiveness of surgical intervention for complete thumb UCL injuries [6,21]. The prospective study reported significant improvements in pain relief, hand function, and overall satisfaction within one year post-surgery, with over two-thirds of patients achieving clinically meaningful gains and 72% reporting high satisfaction. Complementing these findings, Christensen et al. found that even after an average of 24.5 years, most patients remained highly satisfied with their outcomes, despite radiographic osteoarthritis or mild joint instability. Notably, both studies emphasized that objective factors such as surgical timing or imaging findings did not reliably predict patient satisfaction or perceived functional recovery.

Outcomes Across Different Populations

Surgical treatment for complete thumb UCL tears has shown positive results across various groups, including elite athletes, manual laborers, and the general public.

Research targeting athletic populations has emphasized return-to-sport (RTS) and performance outcomes. A study conducted on professional and collegiate athletes undergoing UCL repair with suture tape augmentation reported exceptionally high RTS rates (94-100%) within a brief recovery period, with athletes, particularly MLB players, maintaining their pre-injury performance levels [20,22]. In contrast, some studies have investigated broader populations, including older adults and individuals undergoing tendon autograft reconstruction for chronic UCL injuries. Their findings revealed favorable outcomes, though recovery periods were longer and mild long-term issues such as stiffness or chronic pain were more prevalent. Despite these challenges, patients reported high satisfaction levels and notable improvements in hand strength and functionality [21,23]. These findings underscore the adaptability and effectiveness of surgical interventions across varied patient needs, with technique selection, timing of surgery, and rehabilitation protocols best guided by individualized patient factors such as age, activity demands, tissue quality, and injury chronicity to maximize long-term functional success and patient satisfaction.

A retrospective study presented strong evidence that thumb UCL repair with suture tape augmentation allows competitive athletes to return to play quickly and safely, with all participants regaining their pre-injury level of performance. However, these findings may not apply to non-athlete groups with different recovery goals and functional needs. While elite athletes benefit from the procedure’s robust biomechanical support and early movement protocols, older or less active individuals may be more concerned with pain reduction and joint stability than rapid rehabilitation. Furthermore, since the study focused solely on high-level athletes, it remains unclear how effective this technique is for recreational athletes or manual laborers, whose job requirements and access to specialized postoperative care may vary considerably [20]. These distinctions underscore the need for further research to evaluate this technique’s efficacy and functional relevance across more diverse patient groups.

The study by Barrera-Ochoa et al. (2024) compared BLR and NBLR, demonstrating comparable short-term outcomes in terms of stability, ROM, and strength. However, the applicability of these outcomes may differ among various groups, including athletes, older adults, and individuals with physically demanding jobs. For instance, athletes or laborers who need to regain function quickly might find NBLR more advantageous due to its shorter immobilization period and accelerated rehabilitation timeline. Conversely, younger patients or those with longer expected lifespans may place greater importance on the long-term durability of BLR. However, the retrospective study did not include extended follow-up to verify this potential benefit. Moreover, since most study participants were male (28 out of 42), the findings may not fully reflect outcomes for females, who may present with different ligament laxity or healing characteristics [19]. Therefore, future studies with larger, more diverse cohorts and extended follow-up periods are needed to better understand the long-term effectiveness and potential gender-related differences in treatment outcomes.

Future Research Priorities

Although there have been significant improvements in the surgical treatment of complete UCL tears of the thumb, essential gaps still exist in the literature. In particular, pediatric patients are underrepresented, with limited research directly comparing surgical outcomes between children and adults. This lack of data raises concerns about how age-related factors may influence ligament healing and the timeline for returning to normal function [17]. The cost-effectiveness of different surgical techniques, including suture anchors and graft reconstructions, is also rarely addressed in detail, limiting evidence-based clinical decision-making [6,21]. Furthermore, there is a lack of high-quality comparative studies evaluating biological versus synthetic grafts, particularly in terms of long-term functional outcomes and complication rates [19,22]. A key methodological issue in many studies is the absence of standardized outcome measures. Although instruments such as the DASH score and grip strength are commonly utilized, the criteria for "success" vary widely and are often inconsistently reported [20,26]. These limitations underscore the need for more rigorous, high-quality prospective studies incorporating standardized outcome measures, diverse patient groups, including pediatric and female populations, and economic assessments. Reaching a consensus on outcome definitions and follow-up durations will be crucial to enhance the comparability and clinical significance of future research.

Limitations

This systematic review followed the PRISMA 2020 guidelines and offers important insights into the surgical treatment of complete UCL tears of the thumb, along with the associated functional and clinical outcomes. However, several limitations must be considered. One significant limitation is the absence of prospective registration in a systematic review database such as PROSPERO, which could compromise transparency and increase the potential for reporting bias. Additionally, many of the included studies had small sample sizes and short follow-up durations, limiting the strength and generalizability of the conclusions. As a result, drawing reliable and broadly applicable insights about surgical outcomes becomes challenging, especially when evaluating uncommon complications or subgroup-specific effects. To strengthen the evidence base, future research should involve larger patient cohorts and extended follow-up periods [17,20,26]. Furthermore, the use of specific inclusion criteria, such as excluding studies published before 2015, non-English language articles, and those with restricted access, may have introduced selection bias. Although the search strategy was thorough and included multiple databases and a wide range of keywords, there remains the possibility that some relevant studies were inadvertently omitted.

The included studies also exhibited considerable variability in surgical techniques, rehabilitation protocols, follow-up durations, and outcome measures, making direct comparisons difficult. Another key limitation is the lack of RCTs among the included studies. The majority were retrospective, observational, or single-center in design. This lack of high-quality evidence limits the ability to draw definitive causal conclusions. These limitations highlight the need for well-designed, multicenter, prospective studies with standardized methodologies to provide stronger, more reliable guidance for clinical practice.

## Conclusions

According to this systematic review, surgical treatment remains the gold standard for complete UCL tears of the thumb, especially in the presence of Stener lesions, joint instability, or unsuccessful conservative management. Various surgical methods, such as suture anchor repair, tendon graft reconstruction, and internal brace augmentation, have consistently shown positive results, with the choice of technique depending on factors such as injury chronicity, tissue condition, and the patient’s activity level. Across multiple studies, functional and clinical outcomes reliably demonstrate restored grip and pinch strength, reduced pain, and high patient satisfaction, even in chronic cases. However, earlier intervention typically leads to better outcomes. Patient-reported outcomes indicate lasting satisfaction and perceived hand function, regardless of minor residual laxity or radiographic findings. Though recovery times and expectations may vary, these surgical approaches have proven effective in diverse populations, including elite athletes, manual laborers, and older adults. While biological and synthetic reconstruction techniques offer similar short-term success, their long-term effectiveness may differ based on individual patient needs. This review emphasizes that choosing the surgical approach for complete UCL tears of the thumb should be tailored to each patient, considering factors such as injury duration, tissue quality, and activity demands, to achieve optimal functional recovery. Furthermore, there is a strong need for rigorously designed RCTs to develop standardized protocols for surgical indications, techniques, and postoperative management. Conducting such studies would improve the consistency of treatment outcomes and provide clearer evidence to support clinical decisions across a wide range of patient populations.
